# Level of Physical Activity and In-Hospital Course of Patients with Acute Coronary Syndrome

**DOI:** 10.5935/abc.20160006

**Published:** 2016-01

**Authors:** Juliana de Goes Jorge, Marcos Antonio Almeida Santos, José Augusto Soares Barreto Filho, Joselina Luzia Menezes Oliveira, Enaldo Vieira de Melo, Norma Alves de Oliveira, Gustavo Baptista de Almeida Faro, Antônio Carlos Sobral Sousa

**Affiliations:** 1 Núcleo de Pós-graduação em Ciências da Saúde pela Universidade Federal de Sergipe, Aracaju, SE - Brazil; 2 Centro de Ensino e Pesquisa do Hospital e Fundação São Lucas, Aracaju, SE - Brazil; 3 Departamento de Medicina da Universidade Federal de Sergipe, Aracaju, SE - Brazil

**Keywords:** Acute Coronary Syndrome / mortality, Risk Factors, Sedentary Lifestyle, Questionnaires, Motor Activity / physiology, Exercise

## Abstract

**Background:**

Acute coronary syndrome (ACS) is one of the main causes of morbidity and mortality in the modern world. A sedentary lifestyle, present in 85% of the Brazilian population, is considered a risk factor for the development of coronary artery disease. However, the correlation of a sedentary lifestyle with cardiovascular events (CVE) during hospitalization for ACS is not well established.

**Objective:**

To evaluate the association between physical activity level, assessed with the International Physical Activity Questionnaire (IPAQ), with in-hospital prognosis in patients with ACS.

**Methods:**

Observational, cross-sectional, and analytical study with 215 subjects with a diagnosis of ACS consecutively admitted to a referral hospital for cardiac patients between July 2009 and February 2011. All volunteers answered the short version of the IPAQ and were observed for the occurrence of CVE during hospitalization with a standardized assessment conducted by the researcher and corroborated by data from medical records.

**Results:**

The patients were admitted with diagnoses of unstable angina (34.4%), acute myocardial infarction (AMI) without ST elevation (41.4%), and AMI with ST elevation (24.2%). According to the level of physical activity, the patients were classified as non-active (56.3%) and active (43.7%). A CVE occurred in 35.3% of the cohort. The occurrence of in-hospital complications was associated with the length of hospital stay (odds ratio [OR] = 1.15) and physical inactivity (OR = 2.54), and was independent of age, systolic blood pressure, and prior congestive heart failure.

**Conclusion:**

A physically active lifestyle reduces the risk of CVE during hospitalization in patients with ACS.

## Introduction

Acute coronary syndrome (ACS) is one of the main causes of death in the modern world^1^ and accounts for about 30% of the deaths in Brazil and 10% of the hospital admissions covered by the Brazilian Unified Health System (*Sistema Único de Saúde*).^2^

The high incidence of ACS occurs due to physical inactivity and failure in the control of classic risk factors (RF) such as smoking, hypertension, diabetes mellitus (DM), dyslipidemia, and obesity.^3,4^ According to a survey conducted by the Brazilian Society of Cardiology, 85% of the Brazilian population is physically inactive.^5^

The specific mechanisms by which physical activity and physical conditioning reduce mortality remain uncertain. Physical activity is associated with favorable changes in cardiovascular risk, since it reduces obesity, improves body fat distribution, and decreases the incidence of type 2 diabetes mellitus. Regular exercise also has a modest but beneficial effect on blood pressure and lipoprotein profile.^6,7^

New evidence suggests that exercise training promotes favorable changes in the fibrinolytic system, autonomous nervous system, and endothelial function, leading to modifications that may influence the cardiovascular function and reduce the cardiovascular risk. It has also been shown that exercise training in individuals with known coronary disease improves myocardial perfusion and reduces atherosclerosis progression and levels of myocardial ischemia in response to a certain degree of effort. Data suggest that the perfusion effect may improve the clinical outcome and reduce cardiac events, resulting in a better outcome after acute myocardial infarction (AMI).^7^

Despite the high incidence, morbidity, and mortality of ACS and the recognized protective role of regular practice of physical activity in AMI prevention, the inverse correlation between physically active lifestyle and occurrence of cardiovascular events (CVE) during hospitalization of patients with ACS is not well established. Therefore, the aim of this study was to evaluate with the International Physical Activity Questionnaire (IPAQ) the degree of physical activity of patients with ACS and its association with in-hospital prognosis.

## Methods

### Study Type

Observational, cross-sectional, and analytical study.

### Patients and Methods

Nonrandom convenience sample recruited consecutively. The sample size was estimated based on the frequency of admission of patients with ACS to the Chest Pain Unit (CPU) in a hospital considered a referral center for patients with cardiac disease in Sergipe, Brazil. This hospital has received a Level 3 Accreditation (IQG - *Instituto Qualisa de Gestão*) and is part of the SOLAR (*São Lucas Registro em SCA*) registry.^8^ The estimated minimum sample size was 195 individuals and was based on the following parameters: two-tailed alpha = 0.05, power = 90%, probability of an event in the group with greater physical activity = 30%, odds ratio (OR) of the less active *versus* the more active group = 1.7. We increased in 10% the sample size due to a potential size reduction if some individuals decided to withdraw the research consent. Considering an approximate rate of 10 admissions per month, we then obtained a final sample size of 215 patients.

We recruited patients of both genders admitted with ACS to the CPU of the hospital mentioned above between July 2009 and February 2011. All patients were covered by supplemental health insurance.

All volunteers included in the study answered the short version of the IPAQ. The questionnaire was applied individually after admission of the patient to the CPU. The participants received instructions and recommendations to complete the questionnaire without a time limit. Any questions by the patients were promptly clarified by the investigator, who personally accompanied all the data collection.

### Inclusion and Exclusion Criteria

The study included patients with ACS (unstable angina, non-ST-elevation myocardial infarction [non-STEMI], or ST-segment elevation myocardial infarction [STEMI]), determined by clinical history (symptoms consistent with acute ischemia) and serial increase in markers of cardiac necrosis, and confirmed by at least one of the following tests: electrocardiography, transthoracic Doppler echocardiography, and coronary cineangiography. The only exclusion criterion was the inability to complete the questionnaire (ex: hemodynamic instability, dementia, delirium, severe depressive disorders, etc).

### Patients

To determine the clinical and laboratory profile and the in-hospital course of the ACS patients, we performed a standardized assessment implemented by the researcher and corroborated by data from medical records. The following parameters were evaluated: a) patient identification; b) clinical condition on admission (diastolic blood pressure [DBP], systolic blood pressure [SBP], heart rate [HR]); c) treatment in the acute phase (percutaneous coronary intervention with stent implantation, transluminal balloon angioplasty, coronary artery bypass grafting, and pharmacological treatment); d) routine tests (blood count, creatinine, blood glucose, urea, serum lipid profile, sodium and potassium, markers of myocardial necrosis [troponin, CK-MB], echocardiography); e) medical history and cardiovascular RF (hypertension, DM, dyslipidemia, prior and current smoking, prior cardiovascular diseases, etc.), f) anthropometric measurements (weight and height for calculation of body mass index [BMI]), and g) in-hospital occurrence of CVE (cardiovascular death, recurrent ischemic events, acute pulmonary edema [APE], stroke, cardiogenic shock, and atrial fibrillation), and length of hospital stay.

We defined as smokers those patients who maintained the habit of smoking, and as ex-smokers those who had quit smoking for at least one year. Patients were classified as diabetics if they had a previous diagnosis of DM and/or were using hypoglycemic agents, or if they had a fasting blood glucose > 126 mg/dL in a previous test or during hospitalization. They were considered hypertensive if they already had a diagnosis of hypertension prior to the admission, and/or were using antihypertensive medications, or if they presented systolic blood pressure (SBP) ≥ 140 mmHg and/or diastolic blood pressure (DBP) ≥ 90 mmHg. Dyslipidemia was determined by the occurrence of increased serum levels of LDL-C and/or decreased serum levels of HDL-C and/or increased serum levels of triglycerides (LDL-C > 130 mg/dL, HDL-C < 40 mg/dL, and triglycerides > 150 mg/dL). Patients with BMI > 25 kg/m^2^ were considered to have excess weight.^9^

A diagnosis of recurrent myocardial ischemia was based on recurrent ischemic symptoms, new electrocardiographic changes, and/or a new elevation in CK-MB levels after a decrease from a peak value.^10,11^ APE was defined as the presence of clinical signs of left ventricular failure, dyspnea, and signs of hypoxia and fluid in the lungs (that is, observation of crackles on pulmonary auscultation and bilateral pulmonary infiltrates consistent with congestion on chest x-ray). Stroke was defined as the rapid development of clinical signs of focal (or global) brain function disorder lasting more than 24 hours without an apparent cause other than vascular.^12^ Cardiogenic shock was determined in the presence of hypotension (SBP < 90 mmHg or 30 mmHg below the baseline value), evidence of tissue hypoperfusion such as oliguria, cyanosis, cold extremities, and changes in consciousness level, pulmonary capillary pressure > 18 mmHg, cardiac index < 1.8 L/min/m^2^, systemic vascular resistance index > 2,000 dyne.sec/cm^5^/m^2^ and increase in O_2_ arteriovenous difference > 5.5 mL/dL.^13-15^

The IPAQ, the questionnaire used in our study, collects information regarding the routine practice of physical activity. This questionnaire has been proposed by the Consensus Group for the Development of an International Physical Activity Questionnaire, formed under the seal of the World Health Organization and with representatives in 25 countries, including Brazil.^16^ We chose the short form of this self-administered questionnaire (version 8), which is composed of eight open questions investigating activities in the prior week. The questions explore the frequency (days/week) and time (minutes/day) dedicated to walking and to activities involving physical effort of moderate and vigorous intensities, in addition to activities performed in the sitting position. Vigorous physical activities are those that require major physical effort and breathing much more intense than normal, whereas moderate physical activities are those that require some physical effort and breathing a little more intense than the normal.

To classify the routine practice of physical activity, we followed the consensus proposed by the *Centro de Estudos do Laboratório de Aptidão Física de São Caetano do Sul* (a center that oversees the implementation of the IPAQ in Brazil), and considered four strata:^17^ I - *Very Active:* ≥ 30 minutes/session of vigorous activity ≥ 5 days/week; and/or ≥ 20 minutes/session of vigorous activity ≥ 3 days/week in addition to a ≥ 30 minutes/session of moderate activities or walking ≥ 5 days/week; II - *Active:* ≥ 20 minutes/session of vigorous activity ≥ days/week; and/or ≥ 30 minutes/session of moderate activities or walking ≥ 5 days/week; and/or ≥ 150 minutes/week of any of the added activities (vigorous + moderate + walk); III - *Irregularly Active:* < 150 and > 10 minutes/week of any of the added activities (vigorous + moderate + walk); and IV - *Sedentary:* ≤ 10 minutes/week of any of the added activities (vigorous + moderate + walking).

Based on this stratification and experience from clinical practice, the subjects were classified into two groups: non-active, comprising sedentary and irregularly active individuals; and active, encompassing active and very active individuals.

It is important to emphasize that we considered a patient as practicing physical activity when he or she exercised regularly for at least 3 months before the first SCA event.

### Ethical Aspects

The study was approved by the Ethics Committee in Research of UFS (*Universidade Federal de Sergipe*) with the number 5673.0.000.107-09. Prior to participating in the study, all volunteers signed a free and informed consent form.

### Statistical Analysis and Data Interpretation

The qualitative variables were expressed as frequency (percentage), and the quantitative variables were analyzed with the Kolmogorov-Smirnov test to determine the type of distribution; those which met the assumption of normality were presented as mean and standard deviation. The variables without a normal distribution were described as median and interquartile intervals or minimum and maximum values.

For comparisons between qualitative variables, we used the chi-square test or Fisher's exact test, when appropriate. We used the Student *t* test for comparisons between the two main groups when the continuous or discrete variables had a normal distribution. Variables with asymmetric distribution were analyzed with the Mann-Whitney test.

The association between level of physical activity and occurrence of in-hospital complications was evaluated with logistic regression to determine unadjusted and adjusted odds ratios. For inclusion in the model, we considered crude odds ratio with p < 0.10, and to remain in the model, p < 0.05.

All analyses were performed using SPSS, version 17.0. The differences observed during the analyses were considered statistically significant when the probability was < 0.05.

## Results

### Characteristics of the Study Population

We evaluated 215 volunteers with a mean age of 66.5 ± 14.7 years, of whom 124 (57.7%) were male and 124 (57.7%) were Caucasians. Regarding the type of ACS, 34.4% of the individuals had unstable angina, 41.4% had non-STEMI, and 24.2% had STEMI. According to the level of physical activity, patients were classified as non-active (n = 121, 56.3%), or active (n = 94, 43.7%). Table 1 shows the baseline characteristics of the population.

**Table 1:** Baseline characteristics of patients with ACS

	**Physical Activity Level**			
**Variable**	**General (n = 215)**	**Non-active (n = 121)**	**Active (n = 94)**	**p**
**Demographic data**				
Age (years), mean ± SD	66.5 ± 14.7	68.2 ± 15.8	64.3 ± 12.8	0.05
Male Gender, n (%)	124 (57.7)	70 (57.9)	54 (57.4)	1.00
Caucasians, n (%)	124 (57.7)	77 (63.6)	47 (50)	0.05
**Admission data, mean ± SD**				
Systolic Blood Pressure (mmHg)	137 ± 26.3	140.1 ± 28.7	132.8 ± 22.4	0.04*
Diastolic Blood Pressure (mmHg)	80.7 ± 14.8	81.7 ± 16.3	79.4 ± 12.7	0.24
Heart Rate (bpm)	79.3 ± 16.9	80.7 ± 18.2	77.5 ± 14.9	0.17
Ejection Fraction (%)	58 ± 12	56 ± 12	62 ± 10	< 0.001*
**Diagnosis, n (%) 0.28**				
Acute Myocardial Infarction with ST elevation	52 (24.2)	25 (20.7)	27 (28.7)	-
Acute Myocardial Infarction without ST elevation	89 (41.4)	55 (45.5)	34 (36.2)	-
Unstable Angina	74 (34.4)	41 (33.9)	33 (35.1)	-

(*) p ≤ 0.05; n: Number; SD: Standard deviation; mmHg: Millimeters of mercury; bpm: Beats per minute; %: Percentage; ACS: Acute coronary syndrome.

At baseline, there were no differences between the groups regarding age, gender, BMI, DBP, HR, and type of ACS. However, non-active individuals presented significantly lower ejection fraction (EF, p < 0.001) and higher mean SBP (p = 0.04) than active individuals (Table 1).

With regard to prior cardiovascular diseases, only congestive heart failure (p = 0.002) and deep venous thrombosis (p = 0.02) were more prevalent in the non-active compared with the active group. Regarding the occurrence of cardiovascular RF, approximately 3/4 of the patients were hypertensive, about 1/3 had dyslipidemia, a few less than half of the patients had DM and approximately 1/4 were obese and smokers, without significant differences between the groups (Table 2).

**Table 2:** Medical history and cardiovascular risk factors in patients with ACS

	**Physical Activity Level**			
**Variable**	**General (n = 215)**	**Non-active (n = 121)**	**Active (n = 94)**	**p**
**Medical History, n (%)**				
Family History of Obstructive Heart Failure	116 (54)	68 (31.6)	48 (22.3)	0.49
Prior Coronary Artery Disease	102 (47.4)	60 (49.2)	42 (44.7)	0.49
Prior Acute Myocardial Infarction	62 (28.8)	39 (32.2)	23 (24.5)	0.22
Unstable Angina	122 (56.7)	71 (58.7)	51 (54.3)	0.57
Stable Angina	29 (13.5)	13 (10.7)	16 (17)	0.22
Prior Transluminal Coronary Angioplasty	96 (44.7)	53 (43.8)	43 (45.7)	0.78
Prior Myocardial Revascularization	41 (19.1)	24 (19.8)	17 (18.1)	0.86
Dyslipidemia	130 (60.5)	72 (59.5)	58 (61.7)	0.78
Diabetes Mellitus	95 (44.2)	56 (46.3)	39 (41.5)	0.49
Hypertension	160 (74.4)	90 (74.4)	70 (74.5)	1.00
Smoking	40 (18.6)	23 (19)	17 (18.1)	1.00
Prior Congestive Heart Failure	44 (20.5)	34 (28.1)	10 (10.6)	0.002*
Prior Arrhythmia	67 (31.2)	42 (34.7)	25 (26.6)	0.23
Prior Deep Venous Thrombosis	24 (11.2)	19 (15.7)	5 (5.3)	0.02*
Prior Stroke	23 (10.7)	16 (13.2)	7 (7.4)	0.19
Body Mass Index, mean ± SD	26.9 ± 4.4	26.8 ± 4.4	27.0 ± 4.5	0.71

(*) p ≤ 0.05; n: Number; SD: Standard deviation; %: Percentage; ACS: Acute coronary syndrome.

### Patients' In-hospital Course

**Figure 1:**
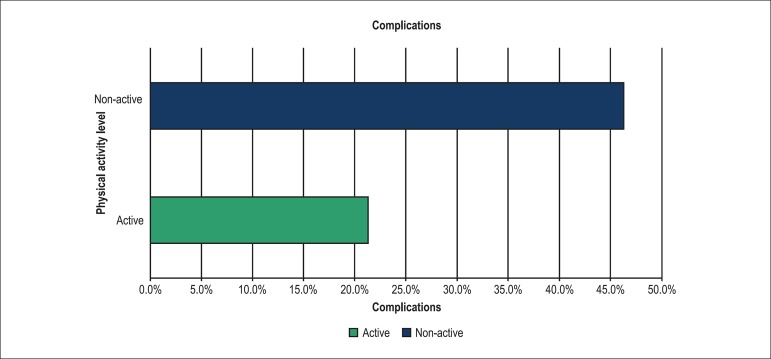
Frequency of complications during hospital stay in patients with ACS.

A CVE occurred in 35.3% of the cohort, with an increasing trend in the frequency of complications with a decrease in physical activity level (p < 0.001) (Figure 1). The characteristics of the patients' in-hospital course are shown in Table 3.

We observed a significant difference in the frequency of APE (p = 0.01), stroke (p = 0.03), and atrial fibrillation (p = 0.01) among patients with different levels of physical activity (Table 3). However, the groups did not show differences regarding the frequency of cardiogenic shock (p = 0.13), reinfarction (p = 0.45), or death (p = 1.00). As for the length of hospital stay, there was a difference between the activity levels, with medians of 8 days (interquartile range = 5-10 days) for the less active group and 6 days (interquartile range = 4-8 days) for the more active group. This difference was significant when analyzed by the Mann-Whitney test (p = 0.011).

**Table 3:** Outcome of patients with ACS

	**Physical Activity Level**			
**Variable**	**General (n = 215)**	**Non-active (n = 121)**	**Active (n = 94)**	**p**
**Cardiovascular Events**				
Cardiovascular Events, n (%)	76 (35.3)	56 (46.3)	20 (21.3)	< 0.001 *
Acute Pulmonary Edema, n (%)	30 (14)	23 (19)	07 (7.4)	0.01 *
Reinfarction, n (%)	35 (16.3)	22 (18.2)	13 (13.8)	0.45
Stroke, n (%)	6 (2.8)	6 (5)	0 (0)	0.03 *
Shock, n (%)	4 (1.9)	2.3 (3.3)	1.7 (0.0)	0.13
Atrial Fibrillation, n (%)	12 (5.6)	11 (9.1)	1 (1.1)	0.01 *
Death, n (%)	3 (1.4)	2 (1.7)	1 (1.1)	1.00

(*) p ≤ 0.05; n: Number; %: Percentage; ACS: Acute coronary syndrome.

The odds ratio of a non-active patient presenting a complication when compared with an active patient was 2.54 (95% confidence interval [CI] 95% = 1.24 - 5.20; p = 0.01) (Table 4). Finally, the occurrence of in-hospital complications had a stronger association with physical inactivity (OR = 2.54) than with length of hospital stay (OR = 1.15) in a multivariate analysis that included the variables physical activity level, age, gender, diagnosis, EF, DM, hypertension, smoking, BMI, and length of hospital stay (Table 4).

**Table 4:** Adjusted odds ratio for cardiovascular complications related to the level of physical activity

**Variable**	**OR**	**95% CI**	**p**
**Physical activity level**			
Non-active	2.54	1.24-5.20	0.01*
Active	1		
Age	0.99	0.97-1.02	0.87
**Gender**			
Male	1.77	0.85-3.67	0.12
Female	1		
**Diagnosis**			
Acute Myocardial Infarction with ST Elevation	1.25	0.47-3.26	0.64
Acute Myocardial Infarction without ST Elevation	1.92	0.84-4.38	0.11
Unstable Angina	1		
Ejection Fraction	0.108	0.00-2.10	0.14
**Diabetes Mellitus**			
Yes	1.33	0.65-2.73	0.42
No	1		
**Hypertension**			
Yes	1.14	0.52-2.52	0.73
No	1		
**Smoking**			
Yes	2.13	0.86-5.23	0.09
No	1		
Body Mass Index	0.93	0.86-1.00	0.07
Length of Hospital stay	1.15	1.08-1.23	< 0.001*

(*) p ≤ 0.05; OR: Odds Ratio; CI: Confidence Interval; %: Percentage.

The Hosmer-Lemeshow test showed a p value of 0.39 (values higher than 0.05 indicate an adequacy of the model). Similarly, we estimate the classificatory power, which proved to be satisfactory: positive predictive value = 61.54%; negative predictive value = 70.45%; rate of correct classification: 68.84%.

## Discussion

A physically active lifestyle has been associated with a lower risk of ACS.^18^ In this investigation, we also demonstrated its association with a reduction in the occurrence of cardiovascular complications during hospital stay. The results show that sedentary patients when compared with physically active ones had a 2.54 (95% CI 1.24 - 5.20) times greater probability of presenting a recurrent event. In total, 43.7% of the patients were physically active and 35.3% presented CVE during hospitalization.

Data from the multicenter study GREECS (*Greek Study of Acute Coronary Syndromes*), which evaluated the level of physical activity in 2,172 patients with ACS, showed a CVE rate of 9.4%, of which 3.2% were fatal. Of physically inactive patients, 10.6% had an event in the first 30 days after hospitalization. Among the minimally active and very active individuals, 7.1% and 6.3%, respectively, presented events. Analyses adjusted for age and gender showed that physically active patients had a probability 0.8 (95% CI 0.63 - 1.19) times lower of having a recurring event when compared with physically inactive ones.^19^

Examination of similar data sets may help understand our findings. In Brazilian patients enrolled in the GRACE registry,^20^the main in-hospital CVE during a mean length of hospital stay of 9 days were heart failure (21%), reinfarction (15%), cardiogenic shock (11%), death (11%), and stroke (1%). As for the association between CVE and mortality, Jesus et al.,^21^ in a study performed in the same institution as ours, found a CVE rate of 12% during hospitalization, with a mortality rate of 2.5%, which is similar to the findings of the present study.

We found that the frequency of complications during hospital stay in patients with ACS increases as their level of physical activity decreases. These findings are in agreement with those from the GREECS^19^ study which suggest that physical activity is associated with a low risk of death due to a reduction in recurring events. This protective effect may be secondary to a control in RF,^22^ since physical activity reduces oxidative stress, which stabilizes the plaque, and stabilizes cell membranes, which decreases the frequency of arrhythmia.^23,24^

Even though physical activity has a well established cardioprotective effect, the mechanism by which physical exercise exerts this effect, especially in patients with ACS, is not well understood.^7^ Resistance exercises have been associated with a substantial increase in myocardial performance^25^ and infarct extent.^26^ These exercises have the potential to interfere with ischemic preconditioning in the heart since the exercise is itself associated with myocardial ischemia.^27^ The protective effect of ischemic preconditioning occurs in two phases: early (up to 3 hours after the exercise) and delayed (from 24 to 72 hours after the exercise, and possibly related to the increase in cytoprotective proteins).^28^

Ribeiro and Oliveira^29^ stated that regular physical activity is associated with a reduction in the risk of cardiovascular diseases, including a decreased tendency to form thrombi, by reducing coagulation activity and increasing fibrinolytic activity. However, physical exercise increases acutely the coagulation and fibrinolytic responses. In contrast, the chronic effects are positive in individuals in whom these processes are impaired (for example, following an AMI).

Chow et al.^30^ have demonstrated in participants of the OASIS-5 study^31^ that physical activity reduces the early occurrence of CVE, notably AMI, stroke, and mortality. Another potential benefit promoted by regular physical activity is that the adherence to an active lifestyle is by itself a marker of adherence to other beneficial and healthy behaviors.

Some limitations must be considered in the interpretation of our results. The IPAQ is a practical and reproducible instrument, but it adopts an indirect methodology to assess the level of physical activity and is, therefore, subject to flaws. The population of patients in the present study is composed exclusively of beneficiaries of supplementary health insurance and does not include beneficiaries of the Unified Health System. We should also emphasize that the cohort was recruited from a single center. Another potential limitation of this research is the interpretation of the proposed association: the patients may complicate less because they are healthier and practice physical activity, or they may be healthier because they practice physical activity and, therefore, complicate less. Since this is not a study of causality, the research model is unable to advance beyond this point.

Future studies should evaluate the quality of medical care by monitoring the patients after advising them to join a rehabilitation program. This would fulfill one of the goals of observational studies, which is to improve clinical practice.

## Conclusions

We observed a lower frequency of complications during hospital stay in patients with ACS who practiced more physical activity (or were categorized as active). We did not evaluate the frequency in different strata of physical activity levels. The application of the IPAQ to characterize the level of physical activity provides relevant information about the in-hospital prognosis of patients with ACS.

Future studies are required to confirm our data and, more importantly, to test the long-term impact of a routine practice of physical activity on cardiovascular outcomes in patients with ACS.
